# Plasma Levels of Retinol Binding Protein 4 Relate to Large VLDL and Small LDL Particles in Subjects with and without Type 2 Diabetes

**DOI:** 10.3390/jcm8111792

**Published:** 2019-10-25

**Authors:** Hanna Wessel, Ali Saeed, Janette Heegsma, Margery A. Connelly, Klaas Nico Faber, Robin P. F. Dullaart

**Affiliations:** 1 Department of Endocrinology, University of Groningen and University Medical Center Groningen, 9700 RB Groningen, The Netherlands; wessel.hanna@gmail.com; 2 Department of Gastroenterology and Hepatology, University of Groningen and University Medical Center Groningen, 9700 RB Groningen, The Netherlands; a.saeed@umcg.nl (A.S.); j.heegsma@umcg.nl (J.H.); k.n.faber@umcg.nl (K.N.F.); 3 Institute of Molecular Biology and Biotechnology, Bahauddin Zakariya University Multan 66000, Pakistan; 4 Department of Laboratory Medicine, University of Groningen and University Medical Center Groningen, 9700 RB Groningen, The Netherlands; 5 Laboratory Corporation of America Holdings (LabCorp), Morrisville, NC 27560, USA; connem5@labcorp.com

**Keywords:** retinol binding protein 4, retinol, lipoprotein subfractions, large VLDL, small LDL, Type 2 diabetes mellitus, metabolic syndrome, nuclear magnetic resonance spectroscopy

## Abstract

Background: Retinol binding protein 4 (RBP4) carries retinol in plasma, but is also considered an adipokine, as it is implicated in insulin resistance in mice. Plasma RBP4 correlates with total cholesterol, low density lipoprotein (LDL)-cholesterol and triglycerides, and may confer increased cardiovascular risk. However, controversy exists about circulating RPB4 levels in type 2 diabetes mellitus (T2DM) and obesity. Here, we analyzed the relationships of RBP4 and retinol with lipoprotein subfractions in subjects with and without T2DM. Methods: Fasting plasma RBP4 (enzyme-linked immunosorbent assay) and retinol (high performance liquid chromatography) were assayed in 41 T2DM subjects and 37 non-diabetic subjects. Lipoprotein subfractions (NMR spectroscopy) were measured in 36 T2DM subjects and 27 non-diabetic subjects. Physical interaction of RBP4 with lipoproteins was assessed by fast protein liquid chromatography (FPLC). Results: Plasma RBP4 and retinol were strongly correlated (r = 0.881, *p* < 0.001). RBP4, retinol and the RBP4/retinol ratio were not different between T2DM and non-diabetic subjects (all *p* > 0.12), and were unrelated to body mass index. Notably, RBP4 and retinol were elevated in subjects with metabolic syndrome (*p* < 0.05), which was attributable to an association with elevated triglycerides (*p* = 0.013). Large VLDL, total LDL and small LDL were increased in T2DM subjects (*p* = 0.035 to 0.003). Taking all subjects together, RBP4 correlated with total cholesterol, non-HDL cholesterol, LDL cholesterol, triglycerides and apolipoprotein B in univariate analysis (*p* < 0.001 for each). Age-, sex- and diabetes status-adjusted multivariable linear regression analysis revealed that RBP4 was independently associated with large VLDL (β = 0.444, *p* = 0.005) and small LDL particles (β = 0.539, *p* < 0.001). Its relationship with large VLDL remained after further adjustment for retinol. RBP4 did not co-elute with VLDL nor LDL particles in FPLC analyses. Conclusions: Plasma RBP4 levels are related to but do not physically interact with large VLDL and small LDL particles. Elevated RBP4 may contribute to a proatherogenic plasma lipoprotein profile.

## 1. Introduction

The incidence of the metabolic syndrome (MetS) and Type 2 diabetes mellitus (T2DM) has rapidly increased in the past few years [1]. Both MetS and T2DM are associated with an increased risk of cardiovascular disease and reduced life span [2]. Impaired insulin action is a main determinant responsible for the development of T2DM [3]. Hence, continued attention is being paid to the pathogenic mechanisms underlying insulin resistance.

Retinol-binding protein 4 (RBP4) is produced by the liver and adipose tissue and transports retinol (vitamin A) via the circulation to peripheral tissues. However, RBP4 is also considered to be an adipokine, as it has been implicated in the pathogenesis of insulin resistance [4]. In rodent studies, Rbp4 overexpression was convincingly shown to enhance insulin resistance, impair insulin signaling in muscle and induce pathways involved in hepatic gluconeogenesis [4]. Conversely, genetic deletion of Rbp4 in mice was found to enhance insulin sensitivity [4]. Nonetheless, studies in humans with obesity, impaired glucose tolerance and T2DM have shown inconsistent results regarding the associations of these pathologies with circulating RBP4. Initial studies documented that RBP4 levels were elevated in patients with obesity, impaired glucose tolerance and T2DM, and were correlated with HbA1c, fasting glucose and insulin [5]. Enhanced RBP4 levels were also observed in obese children, and (pronounced) weight loss was shown to reduce RBP4 levels in some but not in all studies [6,7,8]. Likewise, studies reporting potential associations between RBP4 and T2DM were also inconsistent. Initial studies observed elevated RBP4 levels in T2DM patients [9,10,11], whereas more recent reports either detected no abnormalities in RBP4 [12] or even found a negative correlation of RBP4 with diabetes status [13]. RBP4 is the major carrier of retinol in plasma although not all RBP4 may be “loaded” with retinol. The RBP4/retinol ratio was found to be elevated in T2DM despite lower RBP4 levels as such [13], and similar observations were made in obese children [14]. The RBP4/retinol ratio has therefore been advocated to better reflect alterations in obesity and T2DM [13,14].

In contrast to the equivocally reported relationships of circulating RBP4 with obesity, dysglycemia and insulin resistance, positive relationships of RBP4 with total cholesterol, low density lipoprotein (LDL) cholesterol and triglycerides have repeatedly and almost invariably been demonstrated [6,7,12,13,15,16,17]. Quantification of specific lipoprotein subfractions is increasingly used to better delineate dyslipidemia in pathological conditions, such as obesity and (pre)diabetes [18,19,20,21]. Interestingly, RBP4 associates with small dense LDL particles and oxidized LDL [17,22]. No data are currently available regarding the relationship of RBP4 with nuclear magnetic resonance (NMR)-quantified lipoprotein subfractions in adult subjects with and without T2DM, the latter condition being expected to be characterized by elevated large VLDL and small-sized LDL particles [18,20]. Given that an increasing number of human studies suggest that elevated plasma RBP4 levels may confer (subclinical) cardiovascular disease (CVD) risk [15,23,24,25,26], it is clinically relevant to more precisely delineate the association of RBP4 with various lipoprotein subfractions.

Therefore, the present study aimed to determine whether plasma levels of RBP4 and/or retinol relate to VLDL and LDL subfractions in adults with and without T2DM.

## 2. Experimental Section

### 2.1. Subjects

The study protocol was approved by the medical ethics committee of the University Medical Center Groningen. All participants provided written informed consent and were aged > 18 years. They were recruited by advertisement in local newspapers. T2DM was diagnosed by primary care physicians based on fasting plasma glucose ≥ 7.0 mmol/L and/or a non-fasting plasma glucose ≥ 11.1 mmol/L. Diabetic patients using metformin, sulfonylurea and antihypertensives were included. Insulin use was an exclusion criterion. A positive history of cardiovascular disease, chronic kidney disease (estimated glomerular filtration rate < 60 mL/min/1.73 m^2^ and/or proteinuria), abnormal liver function tests (transaminases > 3 times the upper reference limit) or thyroid dysfunction (thyroid stimulating hormone > 10 or < 0.40 mU/L or use of thyroid function influencing medication), as well as current smoking and use of lipid lowering drugs was also an exclusion criterion. Blood pressure was measured after 15 min of rest in the left arm in sitting position using a sphygmomanometer. Body mass index (BMI in kg/m^2^) was calculated as weight divided by height squared. Waist circumference was measured on bare skin between the 10th rib and the iliac crest. The participants were studied after an overnight fast. Metabolic syndrome (MetS) was defined according to NCEP-ATP III criteria [27]. Three or more of the following criteria were required for categorization of subjects with MetS: waist circumference > 102 cm for men and > 88 cm for women; hypertension (blood pressure ≥ 130⁄85 mmHg or use of antihypertensive drugs); fasting plasma triglycerides ≥ 1.70 mmol/L; HDL cholesterol <1.00 mmol/L for men and <1.30 mmol/L for women; fasting glucose ≥ 5.60 mmol/l. Insulin sensitivity was estimated by homeostasis model assessment of insulin resistance (HOMA-IR) applying the following equation: fasting plasma insulin (mU/L) × glucose (mmol/L)/22.5.

### 2.2. Laboratory Analysis

Serum and EDTA-anticoagulated plasma samples were stored at −80 ℃ until analysis. Plasma glucose was measured shortly after blood collection with an APEC glucose analyzer (APEC Inc., Danvers, MA, USA). Plasma total cholesterol and triglycerides were measured by routine enzymatic methods (Roche/Hitachi cat. nos 11,875,540 and 1,187,602, respectively; Roche Diagnostics GmBH, Mannheim, Germany). HDL cholesterol was assayed by a homogeneous enzymatic colorimetric test (Roche/Hitachi, cat.no 04713214). Non-HDL cholesterol was calculated as the difference between total cholesterol and HDL cholesterol. LDL cholesterol was calculated by the Friedewald formula if triglycerides were <4.5 mmol/L. Apolipoprotein A-I (apoA-I) and apolipoprotein B (apoB) were measured by immunoturbidimetry (Roche/Cobas Integra Tinaquant cat no. 03032566, Roche Diagnostics). HbA1c was measured by high-performance liquid chromatography (HPLC; Bio-Rad, Veenendaal, the Netherlands; normal range: 27–43 mmol/mol).

Plasma RBP4 was assayed by enzyme-linked immunosorbent assay (ELISA; R&D Systems; Catalog Number DRB400). In brief, 96-well plates precoated with anti-human RBP4 monoclonal antibody were stored at 4 °C. After adding 200 μL of 2 vials (11 mL/vial) of a buffered protein solution to each well, 20 μL of sample material was added to each well and left for 1 h at room temperature on a horizontal orbital microplate shaker (0.12” orbit) set at 500 rpm. This procedure was followed by aspiration and washing of each well with 400 μL buffered surfactant, repeating the process three times for a total of four washes. 200 μL of monoclonal antibody specific for human RBP4 conjugated to horseradish peroxidase were added to each well and incubated for 1 h at room temperature under gentle agitation. After repeating aspiration/wash as described, 200 μL of Substrate Solution consisting of stabilized hydrogen peroxide and stabilized chromogen (tetramethylbenzidine) were added to each well and incubated for 30 min at room temperature under protection from light. The reaction was then stopped by adding 50 μL of 2 N sulfuric acid; the absorbance was measured at 450 nm, 540 nm and 570 nm.

Plasma retinol levels were analyzed by reverse phase high performance liquid chromatography (HPLC) as described [28]. Briefly, 50 µL plasma samples were added in antioxidant solutions (2.8 mL) and vortexed thoroughly for 1 min. Retinol was extracted and deproteinized twice with n-hexane in the presence of retinol acetate (100 µL, concentration 4 µmol/L) as external standard to assess the level of recovery after the extraction procedure. Standard curves created from a range of concentrations of retinol were used to determine absolute serum retinol concentrations. Additionally, two negative controls (only containing internal standard) and two positive controls (low and high concentrations of retinol plus internal standard) were included in each series of extractions. Samples were evaporated under N2 and diluted in 300 µL 100% ultrapure ethanol. Then, 50 µL were injected in HPLC (Waters 2795 Alliance HT Separations Module, Connecticut, USA) for phase separation on a C18 column (Waters Symmetry C18, dimension 150 × 3.0 mm, particle size 5 µm, Waters Corporation, Milford, MA, USA) (UV-VIS, dual wavelength, UV-4075 Jasco, Tokyo, Japan). Retinoids in samples were identified by applying exact retention time of known standards in ultraviolet absorption at 325 nm by HPLC. Finally, retinol concentrations were calculated and normalized to final volume. The intra-assay coefficients of variation (CV) of RBP4 and retinol amount to 5.7–8.1% and 3.6%, respectively.

Lipoprotein separation was performed by fast protein liquid chromatography (FPLC) as described before [29]. One mL of plasma of four control individuals were subjected to size exclusion gel FPLC filtration using a Superose 6 column (GE Healthcare, Uppsala, Sweden) at a flow rate of 0.5 mL/min. Fractions of 500 µL each were collected. Total protein (Bradford), total cholesterol, triglycerides and RBP4 were determined in the respective lipoprotein fractions by ELISA as detailed above. One representative FPLC profile is presented.

Lipoprotein particle profiles were determined by nuclear magnetic resonance (NMR) spectroscopy with the LP3 algorithm (LipoScience, now LabCorp, Morrisville, NC, USA) [30,31]. Very low density lipoprotein (VLDL), low density lipoprotein (LDL) and high density lipoprotein (HDL) particle classes and subfractions were quantified from the amplitudes of their spectroscopically distinct lipid methyl group NMR signals. Diameter range estimates were for VLDL (including chylomicrons if present): >60 nm to 29 nm, for LDL: 29 nm to 18 nm, and for HDL: 14 nm to 7.3 nm. The VLDL, LDL and HDL particle concentrations (VLDL-P, LDL-P and HDL-P, respectively) were calculated as the weighted average of the respective lipoprotein subclasses. The intra-assay CVs for the lipoprotein parameters are: VLDL-P (11.0%), LDL-P (4.1%), HDL-P (2.0%) and amount to 6.6–27.9%. for the various VLDL, LDL and HDL subfractions.

### 2.3. Statistical Analysis

IBM SPSS software (SPSS, version 24.0, SPSS Inc. Chicago, IL, USA) was used for data analysis. Continuous variables were expressed in means ± SD or medians (interquartile ranges) in case of not-normally distributed data. HOMA-IR, triglycerides and lipoprotein subfractions were log_e_ transformed to achieve approximately normal distributions. Between group differences in variables were determined by T-tests for unpaired data and Chi-square tests where appropriate. Univariate correlations were determined by Pearson correlation coefficients. Multivariable linear regression analyses were carried out to disclose the independent relationships of RBP4, retinol and the RBP4/retinol ratio with either T2DM or MetS. Additionally, multivariable linear regression analyses were performed to disclose the independent associations with RBP4 and retinol with VLDL, LDL and HDL subfraction characteristics, each in separate models. Two-sided *p*-values < 0.05 were considered statistically significant.

## 3. Results

Forty-one (41) subjects with T2DM and 37 subjects without T2DM were included. Clinical characteristics and biochemical data are presented in Table 1. Twenty-nine (29) of the diabetic patients used glucose-lowering drugs (metformin and/or sulfonylurea). Antihypertensive drugs, particularly angiotensin-converting enzyme (ACE) inhibitors, angiotensin II receptor antagonists and diuretics, alone or in combination were used by 19 T2DM subjects; none of the non-diabetic subjects were using antihypertensive drugs. Three non-diabetic women were using estrogens. Other medications were not taken.

**Figure 1:**
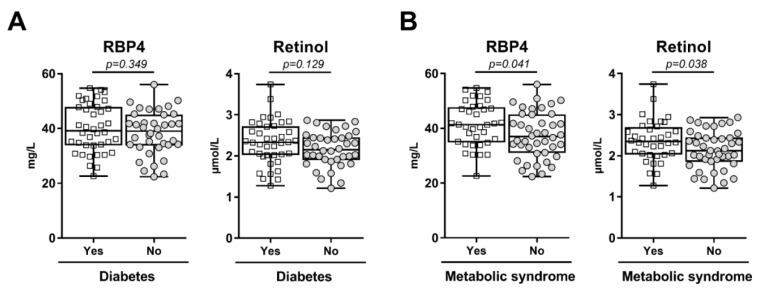
(**A**) Plasma retinol binding protein 4 (RBP4) and retinol in 41 subjects with and 37 subjects without Type 2 diabetes mellitus; (**B**) Plasma retinol binding protein 4 (RBP4) and retinol in 36 subjects with and 42 without the metabolic syndrome (MetS). Data are expressed in box and whiskers plots with mean and minimum to maximum values. All data points are shown.

As expected, T2DM subjects were classified with MetS more frequently, had higher blood pressure, were more obese and had higher glucose and HbA1c levels and had higher HOMA-IR when compared to non-diabetic controls (Table 1). In general, metabolic control was adequate in the diabetic patients given an average HbA1c level of 50 mmol/mol. Total cholesterol, non-HDL cholesterol, LDL cholesterol and apoB levels were not significantly different between diabetic and non-diabetic individuals. Triglycerides were higher, whereas HDL cholesterol was lower in T2DM subjects (Table 1). Plasma levels of RBP4, retinol and the RBP4/retinol ratio were not different between diabetic and non-diabetic subjects (Table 1 and Figure 1A), also not after adjustment for age and sex (RBP4, *p* = 0.63; retinol, *p* = 0.25; RBP4/retinol ratio, *p* = 0,19, respectively; data not shown). In contrast, RBP4 and retinol levels were higher in subjects with MetS vs. subjects without MetS (Figure 1B; RBP4: 41.38 ± 7.98 mg/L in subjects with MetS and 37.48 ± 8.48 mg/L in subjects without MetS, *p* = 0.041; retinol: 2.37 ± 0.50 µmol/L in subjects with MetS vs. 2.14 ± 0.46 µmol/L in subjects without MetS, *p* = 0.038). As a consequence, the RBP4/retinol ratio was not different between subjects with and without MetS (17.61 ± 1.73 mg/µmol vs. 17.57 ± 1.91 mg/µmol, respectively, *p* = 0.78). The associations of RBP4 and retinol with the presence of MetS remained close to significance after adjustment for age and sex (retinol: β = 0.223, *p* = 0.052 and RBP4: β = 0.224, *p* = 0.051; data not shown). Additionally, in age- and sex-adjusted multivariable linear regression analysis, RBP4 was independently associated with elevated triglycerides (β = 0.348, *p* = 0.013), but not with the other individual MetS components (*p* ≥ 0.75 for each). Serum retinol levels were not associated with any of the MetS components (*p* > 0.40 for each; data not shown). In univariate analysis, RBP4 and retinol were not significantly associated with HOMA-IR (r = 0.088, *p* = 0.441 and r = 0.149, *p* = 0.19, respectively). Furthermore, RBP4, retinol and the RBP4/retinol ratio were not significantly different between men and women (RBP4: 39.01 ± 8.40 mg/L in men and 39.43 ± 8.53 mg/L in women, *p* = 0.83; retinol: 2.29 ± 0.46 µmol/L in men and 2.22 ± 0.51 µmol/L in women, *p* = 0.57; RBP4/retinol ratio: 17.10 ± 1.77 mg/µmol in men and 17.90 ± 1.80 mg/µmol in women, *p* = 0.071).

Lipoprotein subfractions were quantified in 36 T2DM subjects and 27 non-diabetic subjects (Table 2). T2DM patients had more large-sized VLDL particles, more LDL particles, more small-sized LDL particles and more small-sized HDL particles, but less large- and medium-sized HDL particles.

**Figure 2:**
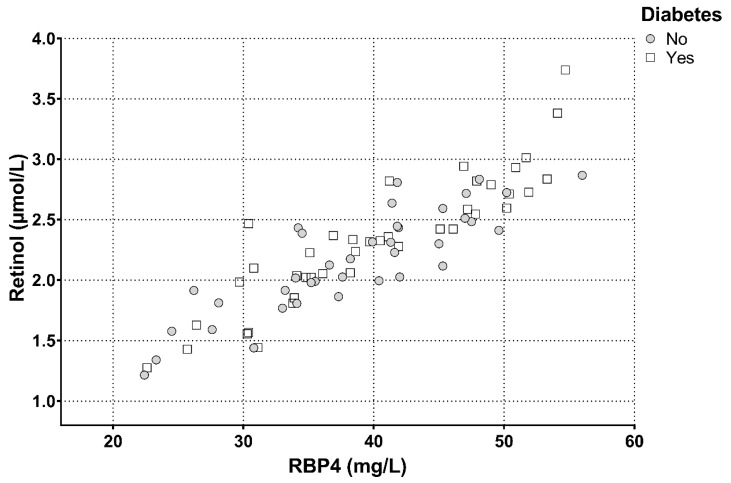
Correlation of total plasma retinol with retinol binding protein 4 (RBP4) in 41 subjects with and 37 without type 2 diabetes mellitus. All subjects: r = 0.881, *p* < 0.001; diabetic subjects: r = 0.900, *p* < 0.001; non-diabetic subjects: r = 0.859, *p* < 0.001.

In univariate regression analysis, RBP4 was strongly correlated with retinol in all subjects combined, and this relationship did not vary according to diabetes status (Table 3A–C). Plasma retinol and RBP4 levels strongly correlated in all subjects combined (r = 0.881, *p* < 0.001), as well as in T2DM subjects (r = 0.900, *p* < 0.001) and non-diabetic subjects separately (r = 0.859, *p* < 0.001) (Figure 2). In all subjects combined, as well as in T2DM subjects and non-diabetic subjects separately, RBP4 and retinol were unrelated to age, blood pressure, obesity measures and glycemic control, except for a positive correlation of RBP4 with age in non-diabetic subjects. Notably, both RBP4 and retinol were positively correlated with total cholesterol, non-HDL cholesterol, LDL cholesterol, triglycerides and apoB levels with essentially similar relationships in T2DM subjects and non-diabetic subjects separately (Table 3A–C).

With respect to lipoprotein subfractions, we observed positive univariate correlations of RBP4 and retinol with VLDL-P, large and medium VLDL, LDL-P, small LDL and small HDL, but inverse correlations with large HDL (Table 4A–C). Again, these relationships were mostly similar in T2DM subjects and non-diabetic subjects separately, although for HDL subfractions these relationships appeared to be stronger in non-diabetic subjects. In addition, large VLDL (r = 0.537, *p* < 0.001), small LDL (r = 0.476, *p* < 0.001) and large HDL (r = −0.403, *p* = 0.001) were correlated with HOMA-IR in all subjects combined. Comparable relationships were found in T2DM and non-diabetic subjects separately (data not shown).

We next performed multivariable linear regression analysis to disclose the extent to which RBP4 and retinol were independently related to VLDL, LDL and HDL subfractions (Table 5). In age-, sex- and diabetes status-adjusted analysis, RBP4 and retinol were independently related to large VLDL and small LDL (Table 5, models A and B). RBP4 and retinol were also independently and inversely associated with large HDL (Table 5, models A and B). When these analyses were additionally adjusted for the use of metformin, sulfonylurea and antihypertensive medication, the positive relationships of large VLDL with RBP4 (β = 0.494, *p* < 0.001) and retinol (β = 0.373, *p* = 0.003), of small LDL with RBP4 (β = 0.511, *p* < 0.001) and with retinol (β = 0.376, *p* = 0.009), and the inverse relationship of large HDL with RBP4 (β = −0.416, *p* = 0.011) and retinol (β = −0.343, *p* = 0.039) remained statistically significant. Remarkably, when retinol was added as an independent variable to the model of RBP4 with VLDL subfractions (Table 5, model A), the relationship of RBP4 with large VLDL remained significant (β = 0.173, *p* = 0.022). Conversely, when RBP4 was added as independent variable in the model of retinol with VLDL subfractions (Table 5, model B), the relation of retinol with large VLDL was lost (β = −0.091, *p* = 0.28).

**Figure 3:**
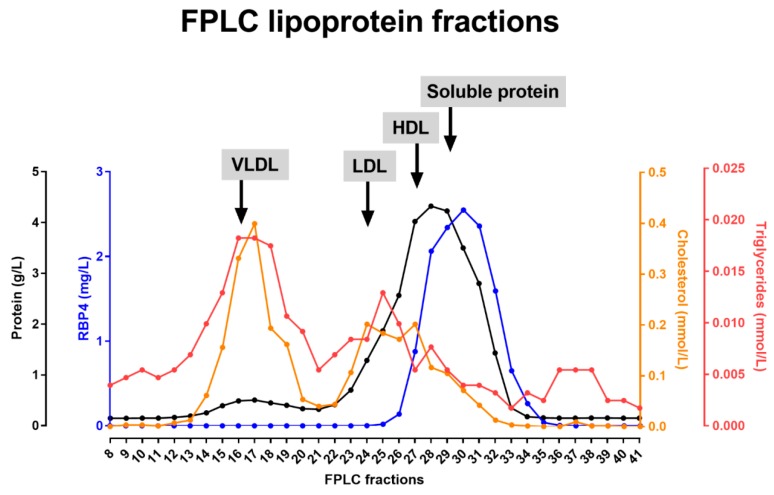
Total protein, retinol binding protein 4 (RBP4), cholesterol and triglyceride concentrations in fast protein liquid chromatography (FPLC) fractions from plasma of a representative control subject. The location of peak fractions of very low density lipoprotein (VLDL), low density lipoprotein (LDL) and high density lipoprotein (HDL) particles, as well as of the soluble proteins are indicated.

Given the correlation of plasma RBP4 levels with certain subfractions, we also assessed whether RBP4 may actually physically interact with such lipoprotein particles. Lipoprotein particles, as well as soluble proteins, were separated by FPLC from freshly-obtained (non-frozen) plasma of non-diabetic individuals and fractions were analyzed for cholesterol, triglycerides, protein and RBP4 concentrations. A representative FPLC profile is shown in Figure 3. RBP4 remained undetectable in the VLDL enriched samples (B1–B9), while very high levels were detected in the fractions with soluble proteins (C2–C10). Some overlap of RBP4 was detected with HDL-containing samples (B10–C2), but RBP4 was absent in the peak fraction of HDL (C1), indicating that the overlap is due to co-elution rather than a physical interaction between RBP4 and HDL. From these experiments, it is concluded that while plasma levels of RBP4 strongly correlate with certain lipoproteins particles; this occurs in the absence of a physical interaction.

## 4. Discussion

This study confirms and extends previous findings [6,7,12,13,16,17], showing that plasma RBP4 is positively correlated with total cholesterol, non-HDL cholesterol, LDL cholesterol, triglycerides and apoB in subjects with and without T2DM. The main novel finding is that the plasma concentration of RBP4 is positively correlated with total VLDL and LDL particles, which appeared to be due to relationships with large VLDL and small LDL particles. The univariate correlations did not differ in diabetic and non-diabetic subjects. In multivariable linear regression analysis adjusted for age, sex, diabetes status and the use of glucose lowering and antihypertensive medication, it was demonstrated that RBP4 was independently related to large VLDL particles, as well as to small LDL particles. The current study thus suggests that the positive relationship of RBP4 with (triglyceride-rich) apoB-containing lipoproteins is attributable to associations with large VLDL and small LDL particles. Notably, no physical interaction appeared to exist between RBP4 and lipoprotein particles.

In our study, plasma RBP4 levels were not elevated in T2DM subjects, in agreement with earlier studies [12,13], but in contrast to other reports [5,9,10,11]. In addition, we did not observe a significant correlation with insulin resistance as determined by HOMA-IR. However, we did find that RBP4 was elevated in subjects classifying with MetS, which was attributable to its association with triglycerides. In comparison, Ingelsson et al. also observed RBP4 elevations in MetS and demonstrated incrementally higher RBP4 levels with an increasing number of MetS components [15]. Of note, while retinol was found to be elevated in the subjects categorized with MetS as well, there was no significant relationship with individual MetS components, and we observed no abnormalities in the RBP4/retinol ratio in T2DM or MetS. In fact, there was a close correlation of retinol with RBP4 as anticipated [14], which appeared to be linear over the full range of RBP4 concentrations observed. Taken together, these findings support the notion that elevated levels of circulating RBP4, rather than retinol, represent the primary abnormality in MetS.

RBP4 levels were predominantly associated with large VLDL particles, as well as with small LDL particles, the latter finding being consistent with earlier observations using conventional methods for lipoprotein subfraction quantification [17]. Given the product-precursor relationships of small LDL particles with large VLDL [18,32,33], it is conceivable that the relationships of RBP4 with these specific VLDL and LDL subfractions are interdependent. We also observed an independent and inverse relationship of RBP4 with large HDL particles. It is well established that HDL cholesterol is inversely associated with large VLDL, consequent to the process of cholesteryl ester transfer [19]. It was recently shown that large VLDL particles determine an increased cholesteryl ester transfer in T2DM [20], but it is unclear whether RBP4 could interfere with this process. Of further interest, RBP4 was associated with large VLDL independent of retinol, but retinol was not associated with large VLDL independent of RBP4. This suggests that circulating RBP4 rather than retinol is primarily related to large VLDL particles. In addition, large VLDL and small LDL particle concentrations were expectedly elevated in T2DM [18,20], again consistent with the concept that large VLDL and small LDL particles are metabolically interrelated [33]. Since our study neither showed an effect of the diabetic state on plasma RBP4 nor on retinol, we surmise that circulating levels of RBP4 and retinol do not to a considerable extent explain the predominance of large VLDL and small LDL particles in this condition. Furthermore, large VLDL and small LDL particles were correlated with HOMA-IR whereas RBP4 and retinol were not. Circulating RBP4 and VLDL particles largely originate from the liver, but their hepatic production and secretion machineries appear not interconnected. Still, the correlation between the circulating levels led us to determine whether a possible physical interaction between RBP4 and VLDL and (small) LDL particles exists. FPLC was used to separate lipoprotein particles from soluble proteins as a mild approach to retain protein-protein and protein-lipid interactions. Still, no co-elution of RBP4 with VLDL was observed, providing no evidence that such physical interaction actually exists. We, therefore, hypothesize that RBP4 could directly interfere with the metabolism of triglyceride-rich apoB-containing lipoproteins. As a potential mechanism, it is of interest that RBP4 transcriptionally enhances CD36 expression [26], a protein that impairs VLDL secretion from mouse liver in vivo [34].

While the current study was primarily focused on RBP4, our findings with respect to retinol levels are also relevant. Retinol is the circulating form of vitamin A that is locally (in tissues) converted to retinoic acids (such as all-trans retinoic acid and 9-cis retinoic acid) that activate transcription factors (Retinoic Acid Receptor (RAR) and Retinoic X Receptor (RXR)) [35,36]. Retinol and retinoic acids simulate hepatic RBP4 secretion and enhance de novo lipogenesis in the liver [34]. In line, retinoic acids also stimulate VLDL secretion in rats [37]. More recently, the enzyme 17-beta hydroxysteroid dehydrogenase 13 (HSD17b13) with retinol dehydrogenase activity, was implicated in the pathogenesis of non-alcoholic fatty liver disease [38]. Retinol may, therefore, play multiple roles in hepatic fat accumulation and triglyceride metabolism, both directly and indirectly, by interacting with RBP4.

In view of the accumulating though sometimes controversial epidemiological evidence indicating that RBP4 is associated with incident CVD [23,24,26,39,40], the present findings with regard to the association of RBP4 with small LDL particles are likely to be clinically relevant. Small dense LDL particles are prone to oxidative modification [17] and may predict incident CVD beyond conventional lipid measures [41,42,43]. Moreover, smaller-sized LDL particles may be more electronegatively charged and upregulate the lectin-like oxidized LDL receptor in vitro, a pro-atherogenic phenomenon [44]. Such particles are associated with the extent and severity of coronary artery atherosclerosis [45]. We, therefore, propose that the relationship of RBP4 with small LDL particles could contribute to its alleged pro-atherogenic potential.

Several methodological issues of the present study need consideration. Diabetic subjects using insulin and lipid lowering were not allowed to participate, in order to obviate confounding due to effects of insulin and lipid-lowering drugs on glucose and lipid metabolism. As a consequence, T2DM subjects with rather mild hyperglycemia and dyslipidemia were preferentially included, which could to some extent mask relationships of RBP4 with dysglycemia and could underestimate relationships of RBP4 with (apo)lipoproteins and lipoprotein subfractions. Furthermore, we excluded subjects with impaired glomerular filtration rate. This was done to minimize confounding with respect to circulating RBP4 due to chronic kidney disease in view of the inverse relationship of RBP4 with glomerular filtration rate [15]. Obviously, the cross-sectional design of our study precludes to establish cause-effect relationships with certainty. Finally, we consider the present findings as preliminary in view of the rather low numbers of diabetic and non-diabetic participants included.

## 5. Conclusions

Serum RBP4 levels are related to large VLDL and small LDL particles, but in the absence of a physical interaction between RBP4 and the lipoprotein particles. Higher RBP4 may be part of a proatherogenic plasma lipoprotein profile. However, it seems unlikely that alterations in circulating RBP4 per se contribute significantly to the predominance of large VLDL and small LDL in the diabetic state.

## Figures and Tables

**Table 1:** Clinical and laboratory characteristics in 41 subjects with Type 2 diabetes mellitus (T2DM) and 37 subjects without T2DM.

	T2DM Subjects (*n* = 41)	Non-Diabetic Subjects (*n* = 37)	*p*-Value
Age (years)	60 ± 10	52 ± 9	<0.001
Gender (men/women)	19/22	10/27	0.127
Metabolic syndrome (yes/no)	29/12	7/30	<0.001
Systolic blood pressure (mm Hg)	145 ± 20	129 ± 20	0.001
Diastolic blood pressure (mm Hg)	87 ± 9	81 ± 12	0.025
BMI (kg/m^2^)	29.0 ± 4.9	25.5 ± 4.1	0.001
Waist (cm)	100 ± 14	84 ± 13	<0.001
Glucose (mmol/L)	8.9 ± 2.3	5.6 ± 0.7	<0.001
HbA1c (mmol/mol)	50 ± 9	33 ± 3	<0.001
HOMA-IR (mU mmol/L^2^/22.5)	4.01 (2.94–6.99)	1.56 (1.13–2.03)	<0.001
Total cholesterol (mmol/L)	5.53 ± 0.97	5.65 ± 0.98	0.578
Non-HDL cholesterol (mmol/L)	4.21 ± 1.05	4.06 ± 1.10	0.547
LDL cholesterol (mmol/L)	3.39 ± 0.88	3.46 ± 0.97	0.73
HDL cholesterol (mmol/L)	1.31 ± 0.39	1.59 ± 0.35	0.002
Triglycerides (mmol/L)	1.90 ± 1.60	1.34 ± 0.53	0.021
ApoB (g/L)	0.97 ± 0.24	0.91 ± 0.26	0.324
Apo A-1 (g/L)	1.37 ± 0.26	1.46 ± 0.21	0.080
RBP4 (mg/L)	40.13 ± 8.71	38.33 ± 8.11	0.349
Retinol (µmol/L)	2.33 ± 0.53	2.16 ± 0.43	0.129
RBP4/retinol ratio (mg/µmol)	17.38 ± 1.70	17.82 ± 1.94	0.290

Data are expressed in means ± SD, medians (interquartile range) or numbers. Abbreviations: Apo: apolipoprotein; BMI: body mass index; HOMA-IR: homeostasis model assessment of insulin resistance; HbA1c: glycated hemoglobin; HDL: high density lipoproteins; LDL: low density lipoproteins; RBP4: retinol binding protein 4. Differences between subjects with and without T2DM were determined by T-tests (using log_e_ transformed values in case of not-normally distributed data) and Chi-square tests where appropriate. LDL cholesterol was calculated in 39 T2DM subjects and in 36 non-diabetic subjects.

**Table 2:** Lipoprotein subfraction characteristics in 36 subjects with Type 2 diabetes mellitus (T2DM) and 27 subjects without T2DM.

	T2DM Subjects (*n* = 36)	Non-Diabetic Subjects (*n* = 27)	*p*-Value
Total VLDL (nmol/L)	68.9 (48.6–82.1)	58.7 (51.4–86.7)	0.56
Large VLDL (nmol/L)	6.8 (2.8–9.9)	2.9 (2.3–4.7)	0.035
Medium VLDL (nmol/L)	23.0 (15.8–40.6)	24.8 (12.5–38.2)	0.45
Small VLDL (nmol/L)	29.9 (20.2–42.9)	32.2 (21.1–44.3)	0.51
Total LDL (nmol/L)	1257 (1022–1540)	981 (856–1284)	0.004
IDL (nmol/L)	170 (118–234)	188 (140–257)	0.14
Large LDL (nmol/L)	509 (345–612)	469 (435–597)	0.57
Small LDL (nmol/L)	586 (400–850)	338 (149–442)	0.003
Total HDL (µmol/L)	32.9 (29.3–37.9)	33.8 (32.0–36.1)	0.75
Large HDL (µmol/L)	5.0 (2.6–6.6)	6.7 (4.6–9.9)	0.021
Medium HDL (µmol/L)	10.1 (8.3–14.4)	12.8 (11.5–16.1)	0.007
Small HDL (µmol/L)	17.7 (14.6–20.9)	14.3 (10.1–16.30	0.006

Data are expressed in medians (interquartile ranges). Abbreviations: VLDL: very low density lipoproteins; LDL low density lipoproteins; IDL: intermediate density lipoproteins; HDL: high density lipoproteins. Differences between subjects with and without T2DM were determined by T-tests using log_e_ transformed values.

**Table 3:** Univariate relationships of retinol binding protein 4 (RBP4) and retinol with clinical and laboratory variables in 78 subjects (**A**), 41 subjects with Type 2 diabetes mellitus (T2DM) (**B**) and 37 subjects without T2DM (**C**).

**All Subjects (A)**	**RBP4**	**Retinol**
RBP4		0.881 ***
Retinol	0.881 ***	
Age	0.153	0.117
Systolic blood pressure	0.151	0.133
Diastolic blood pressure	0.099	0.139
BMI	0.021	0.005
Waist	0.099	0.167
Glucose	0.104	0.196
HbA1c	0.176	0.171
Total cholesterol	0.435 ***	0.399 ***
Non-HDL cholesterol	0.463 ***	0.421 ***
LDL cholesterol	0.384 ***	0.368 ***
HDL cholesterol	−0.186	−0.163
Triglycerides	0.391 ***	0.313 **
ApoB	0.445 ***	0.372 **
ApoA-I	−0.007	−0.039
**T2DM Subjects (B)**		
RBP4		0.859 ***
Retinol	0.859 ***	
Age	−0.082	−0.008
Systolic blood pressure	0.022	0.026
Diastolic blood pressure	0.030	0.065
BMI	−0.117	−0.091
Waist	−0.039	0.030
Glucose	0.082	0.161
HbA1c	0.184	0.089
Total cholesterol	0.376 *	0.321 *
Non-HDL cholesterol	0.425 **	0.340 *
LDL cholesterol	0.315 *	0.271
HDL cholesterol	−0.212	−0.122
Triglycerides	0.415 **	0.293
ApoB	0.394 *	0.262
ApoA-I	−0.150	−0.091
**Non-Diabetic Subjects (C)**		
RBP4		0.900 ***
Retinol	0.900 ***	
Age	0.381 *	0.258
Systolic blood pressure	0.235	0.140
Diastolic blood pressure	0.115	0.144
BMI	−0.117	−0.018
Waist	0.170	0.186
Glucose	−0.100	−0.099
HbA1c	0.051	0.041
Total cholesterol	0.528 ***	0.555 ***
Non-HDL cholesterol	0.500 ***	0.526 ***
LDL cholesterol	0.514 ***	0.463 **
HDL cholesterol	−0.090	−0.096
Triglycerides	0.416 *	0.332 *
ApoB	0.489 ***	0.494 ***
ApoA-I	0.184	0.146

Pearson correlation coefficients are shown. Triglycerides are log_e_ transformed. Abbreviations: BMI: body mass index; HbA1c: glycated hemoglobin; LDL: low density lipoproteins; HDL: high density lipoproteins. * *p* < 0.05; ** *p* ≤ 0.01; *** *p* ≤ 0.001.

**Table 4:** Univariate relationships of retinol binding protein 4 (RBP4) and retinol with lipoprotein subfractions in 63 subjects; (**A**): all subjects; (**B**): 36 subjects with Type 2 diabetes mellitus (T2DM) and (**C**): 27 subjects without T2DM.

**All Subjects (A) (*n* = 63)**	**RBP4**	**Retinol**
Total VLDL	0.452 ***	0.381 **
Large VLDL	0.433 ***	0.341 **
Medium VLDL	0.293*	0.248 *
Small VLDL	0.178	0.187
Total LDL	0.398 ***	0.340 **
IDL	–0.053	–0.098
Large LDL	–0.007	0.118
Small LDL	0.423 ***	0.353 **
Total HDL	0.136	0.126
Large HDL	–0.243	–0.258 *
Medium HDL	–0.056	–0.010
Small HDL	0.317 *	0.290 *
**T2DM Subjects (B) (*n* = 36)**	**RBP4**	**Retinol**
Total VLDP	0.423 *	0.322
Large VLDL	0.401 *	0.262
Medium VLDL	0.238	0.168
Small VLDL	0.160	0.176
Total LDL	0.255	0.191
IDL	–0.227	–0.233
Large LDL	–0.079	0.082
Small LDL	0.351 *	0.266
Total HDL	0.016	0.010
Large HDL	–0.170	–0.190
Medium HDL	–0.004	0.064
Small HDL	0.019	0.002
**Non-Diabetic Subjects (C) (*n* = 27)**	**RBP4**	**Retinol**
Total VLDP	0.487 **	0.472 *
Large VLDL	0.433 *	0.385 *
Medium VLDL	0.344	0.333
Small VLDL	0.270	0.293
Total LDL	0.529 **	0.442 *
IDL	0.309	0.257
Large LDL	0.223	0.301
Small LDL	0.467 *	0.366
Total HDL	0.478 *	0.502 **
Large HDL	–0.287	–0.254
Medium HDL	–0.027	0.051
Small HDL	0.523 **	0.476 *

Pearson correlation coefficients are shown. All lipoprotein subfractions are log_e_ transformed. Abbreviations: VLDL: very low density lipoproteins; LDL: low density lipoproteins; IDL: intermediate density lipoproteins; HDL: high density lipoproteins. * *p* < 0.05; ** *p* ≤ 0.01; *** *p* ≤ 0.001.

**Table 5:** Multivariable regression analyses showing independent relationships of retinol binding protein 4 (RBP4) and retinol with very low density lipoprotein (VLDL) subfractions (**A**), low density lipoprotein (LDL) subfractions (**B**) and high density lipoprotein (HDL) subfractions (**C**) in 63 subjects (36 subjects with and 27 subjects without Type 2 diabetes mellitus).

**A VLDL Subfractions**	**Model A**		**Model B**	
	**β**	***p*****-Value**	**β**	***p*****-Value**
Age	0.305	0.027	0.226	0.12
Sex (men vs. women)	–0.072	0.57	0.018	0.89
T2DM	–0.079	0.60	0.017	0.92
Large VLDL	0.444	0.005	0.324	0.046
Medium VLDL	0.044	0.770	0.032	0.84
Small VLDL	0.183	0.13	0.206	0.105
**B LDL Subfractions**	**Model A**		**Model B**	
	**β**	***p*****-Value**	**β**	***p*****-Value**
Age	0.306	0.035	0.195	0.19
Sex (men vs. women)	–0.180	0.19	–0.099	0.48
T2DM	0.157	0.33	–0.030	0.86
IDL	–0.150	0.24	–0.138	0.30
Large LDL	0.106	0.39	0.221	0.090
Small LDL	0.539	<0.001	0.440	0.003
**B HDL Subfractions**	**Model A**		**Model B**	
	**β**	***p*****-value**	**β**	***p*****-value**
Age	0.309	0.044	0.257	0.095
Sex (men vs. women)	–0.180	0.26	0.006	0.97
T2DM	–0.073	0.65	–0.008	0.96
Large HDL	–0.336	0.034	–0.323	0.043
Medium HDL	0.145	0.38	0.302	0.073
Small HDL	0.262	0.076	0.254	0.086

β: standardized regression coefficient. All lipoprotein subfractions are loge transformed. IDL: intermediate density lipoproteins; **Models A**: RBP4 as dependent variable; **Models B**: retinol as dependent variable.

## Abbreviations

atRA receptor (RAR); apolipoprotein A-I (apoA-I); apolipoprotein B (apoB); body mass index (BMI); cardiovascular disease (CVD); enzyme linked immunosorbent assay (ELISA); fast protein liquid chromatography (FPLC); high density lipoproteins (HDL); high density lipoprotein particle concentration (HDL-P); high performance liquid chromatography (HPLC); homeostasis model assessment of insulin resistance (HOMA-IR); intermediate density lipoproteins (IDL); low density lipoproteins (LDL); low density lipoprotein particle concentration (LDL-P); nuclear magnetic resonance spectrometry (NMR); retinol binding protein 4 (RBP4); very low density lipoproteins (VLDL); very low density lipoprotein particle concentration (VLDL-P)
